# A Portable Extended-Gate FET Integrated Sensing System with Low-Noise Current Readout for On-Site Detection of *Escherichia coli* O157:H7

**DOI:** 10.3390/mi17020151

**Published:** 2026-01-23

**Authors:** Weilin Guo, Yanping Hu, Yunchao Cao, Hongbin Zhang, Hong Wang

**Affiliations:** 1 Guangdong Provincial Key Laboratory of Food Quality and Safety, College of Food Science, South China Agricultural University, Guangzhou 510642, China; 2 Department of Basic Medical Research, General Hospital of Southern Theater Command, Guangzhou 510010, China

**Keywords:** extended-gate field effect transistor, biosensor, on-site detection, *Escherichia coli* O157:H7, immunosensing

## Abstract

Field-effect transistor (FET) biosensors enable label-free and real-time electrical transduction; however, their practical deployment is often constrained by the need for bulky benchtop instrumentation to provide stable biasing, low-noise readout, and data processing. Here, we report a portable extended-gate FET (EG-FET) integrated sensing system that consolidates the sensing interface, analog front-end conditioning, embedded acquisition/control, and user-side visualization into an end-to-end prototype suitable for on-site operation. The system couples a screen-printed Au extended-gate electrode to a MOSFET and employs a low-noise signal-conditioning chain with microcontroller-based digitization and real-time data streaming to a host graphical interface. As a proof-of-concept, enterohemorrhagic *Escherichia coli* O157:H7 was selected as the target. A bacteria-specific immunosensing interface was constructed on the Au extended gate via covalent immobilization of monoclonal antibodies. Measurements in buffered samples produced concentration-dependent current responses, and a linear calibration was experimentally validated over 10^4^–10^10^ CFU/mL. In specificity evaluation against three common foodborne pathogens (*Staphylococcus aureus*, *Salmonella typhimurium*, and *Listeria monocytogenes*), the sensor showed a maximum interference response of only 13% relative to the target signal (ΔI/ΔImax) with statistical significance (*p* < 0.001). Our work establishes a practical hardware–software architecture that mitigates reliance on benchtop instruments and provides a scalable route toward portable EG-FET sensing for rapid, point-of-need detection of foodborne pathogens and other biomarkers.

## 1. Introduction

Rapid and reliable identification of foodborne pathogens is fundamental to outbreak prevention, source tracing, and routine hazard screening in modern food-supply chains [1,2]. In many practical settings—such as production-line inspection, market surveillance, and port-of-entry screening—decision-making is time-critical and therefore requires field-deployable tools that can provide results with minimal delay and operational complexity [3,4]. Among high-risk targets, enterohemorrhagic *Escherichia coli* O157:H7 (*E. coli* O157:H7) is of particular concern because it can initiate severe disease at extremely low infectious doses and is associated with life-threatening complications, including haemolytic uremic syndrome, through Shiga-toxin-mediated vascular injury [5,6,7]. Accordingly, *E. coli* O157:H7 has been designated a key control organism in food safety monitoring frameworks.

A variety of on-site detection platforms have been developed to address this need, each with distinct advantages and limitations. Optical techniques such as fluorescence, surface plasmon resonance (SPR), and localized SPR provide high sensitivity and real-time monitoring, but typically rely on bulky optics, precise alignment, and are particularly susceptible to interference from complex sample matrices like food, which challenges their robustness and true portability for field use [8]. Isothermal nucleic-acid amplification methods, notably loop-mediated isothermal amplification (LAMP), deliver excellent sensitivity and specificity by amplifying target DNA/RNA [9]; however, they involve complex sample preprocessing (e.g., cell lysis, nucleic-acid extraction) and are susceptible to aerosol contamination, making them less suited for rapid, minimally processed sample analysis in resource-limited settings. Even sophisticated research tools like electro-photonic traps for single-bacterium analysis exist [10], but their complexity and cost render them impractical for routine field detection.

Field-effect transistor (FET) biosensors translate biochemical recognition at a solid–liquid interface into an electrical signal by modulating the effective gate potential and, consequently, the channel conductance. This transduction principle supports label-free and real-time readout while retaining the intrinsic advantages of semiconductor devices, including miniaturization, low sample-volume operation, and straightforward integration with electronics, which has motivated extensive use in clinical diagnostics, environmental monitoring, and food-safety screening [11,12,13,14,15,16,17]. However, many FET-based platforms—including those incorporating nanomaterials such as graphene FETs, carbon nanotube (CNT) FETs, and conventional ion-sensitive FETs (ISFETs)—still face practical challenges related to fabrication reproducibility, long-term stability in complex matrices, and integration into compact, low-power readout systems [18,19,20]. Within this family, the extended-gate FET (EG-FET) is particularly attractive for liquid-phase assays because the sensing interface is physically separated from the transistor core: the extended gate can be independently functionalized with biorecognition layers, offers a larger and more flexible surface for surface chemistry, and reduces direct exposure of the transistor to the electrolyte environment, thereby improving operational robustness and device longevity in practical measurements [21,22]. Accordingly, sensitivity and interface stability have been widely optimized through materials and surface engineering (e.g., carbon nanotubes and graphene coatings) to enhance charge-coupling efficiency and interfacial signal transduction [23,24,25,26,27]. Furthermore, this “sensing-readout” separation design provides EG-FET systems with distinct practical features: (1) Low cost—disposable extended gates and commercial FETs can be used, significantly reducing consumable and manufacturing expenses; (2) Easy deployment—compatibility with portable electronics is achieved by optimizing operating modes (e.g., constant-charge OCP mode), eliminating reliance on bulky instruments; (3) Simple sample preparation—well-designed interfacial chemistry enables direct analysis of complex biological samples such as whole blood, saliva, and urine; (4) Fast response—real-time, continuous signal monitoring meets the demand for rapid on-site detection [22]. Therefore, developing integrated EG-FET systems represents a promising pathway toward truly field-ready, practical biosensing platforms.

However, progress at the sensing-element level has not yet translated into equally mature, field-deployable instrumentation. In most reported EG-FET demonstrations, biasing, low-noise current readout, and data processing still rely on bulky benchtop equipment (e.g., semiconductor parameter analyzers, precision source meters, and electrochemical workstations), which undermines portability, increases operational cost, and constrains deployment in decentralized testing scenarios. What remains comparatively underdeveloped is a system-level solution that treats the sensor, analog front-end, embedded acquisition/processing, and user-facing software as a single validated chain, with performance verified in an end-to-end manner under biosensing conditions.

In this work, we develop a compact EG-FET integrated sensing system that combines (i) a self-developed EG-FET sensor array with an antibody-functionalized extended gate for specific capture of *E. coli* O157:H7, (ii) a microcontroller-based acquisition and processing unit enabling real-time signal conditioning and data transmission, and (iii) a host-side visualization interface for data display and basic analysis. As a representative use case, we construct an EG-FET immunosensing interface by covalently immobilizing monoclonal antibodies specific to *E. coli* O157:H7 on the extended-gate surface, enabling selective bacterial capture and a concentration-dependent electrical response. This end-to-end prototype reduces reliance on benchtop instrumentation and provides a practical engineering pathway toward deployable foodborne-pathogen sensing; moreover, the architecture is readily extensible by substituting the recognition layer to target other pathogens (e.g., *Salmonella*, *Listeria*) or food-safety biomarkers (e.g., mycotoxins and antibiotic residues).

## 2. Materials and Methods

### 2.1. Materials and Chemicals

The principal electronic components used to construct the EG-FET integrated sensing system included an n-channel MOSFET (UN600N23TE, UN Semiconductor, Shenzhen, China), a microcontroller unit (MCU, GD32F103C8T6, GigaDevice, Beijing, China), precision operational amplifiers (LTC2051HV, Analog Devices, Wilmington, MA, USA), a USB-to-UART interface (PL2303, Prolific Technology, Shenzhen, China), low-dropout linear regulators (AMS1117, Advanced Monolithic Systems, Livermore, CA, USA), a 3.0 V voltage reference (REF3030, Texas Instruments, Dallas, TX, USA), and screen-printed gold electrodes (G210, Changsha Sanjun Electronic Technology Co., Ltd., Changsha, China).

All chemical and biological reagents were of analytical grade and used as received unless otherwise stated. 3-Mercaptopropionic acid (MPA; M103036, Aladdin, Shanghai, China), 1-ethyl-3-(3-dimethylaminopropyl) carbodiimide hydrochloride (EDC; E106172, Aladdin, Shanghai, China), and N-hydroxysuccinimide (NHS; H109330, Aladdin) were used for surface functionalization. Phosphate-buffered saline (PBS, pH 7.4, C10010500BT, Thermo Scientific, Waltham, MA, USA) served as the working buffer. *E. coli* O157:H7 monoclonal antibody (EC-mab-01, Shanghai Prajna Biology Technique, Shanghai, China) was employed as the biorecognition element, and bovine serum albumin (BSA, BS114, Hefei, China) was used for blocking nonspecific adsorption. A potassium ferricyanide/ferrocyanide redox probe solution (5 mM) was prepared for electrochemical characterization. Ultrapure water was used throughout. The *E. coli* O157:H7 bacterial stock was maintained and preserved in-house.

### 2.2. System Architecture of the EG-FET Integrated Sensing System

#### 2.2.1. Sensing Principle of the EG-FET

The EG-FET separates the sensing interface from the transistor core. The extended gate is functionalized with biorecognition elements (e.g., antibodies) and contacts the electrolyte during measurement. Binding of the target at the gate/solution interface perturbs the interfacial charge distribution and surface potential, producing an effective gate-voltage shift (ΔV_GS_), which is capacitively coupled to the MOSFET gate through the conductive connection, modulating channel conduction. This perturbation is capacitively coupled to the MOSFET gate through the conductive connection, modulating channel conduction. The biochemical recognition event is therefore converted into an electrical readout, typically recorded as a change in drain–source current (I_DS_) at fixed bias or as an equivalent shift in the apparent threshold condition.

#### 2.2.2. Overall System Design

The EG-FET integrated sensing system is implemented as an end-to-end chain comprising a sensor module, an analog front-end for biasing and low-noise current readout, an MCU-based acquisition/communication unit, and host-side software for visualization and data management. The analog front-end converts the weak sensor current into a voltage signal and conditions it for digitization. The MCU (GD32F103C8T6, GigaDevice, Beijing, China) coordinates sampling, performs basic digital processing, and transmits data via a UART link. A host computer interface receives and parses the data stream, displays real-time traces, and stores measurement records for subsequent analysis. Power delivery and voltage referencing are integrated to support stable operation of both analog and digital blocks.

### 2.3. Hardware Design

#### 2.3.1. Current Sampling and Signal Conditioning Module

Weak-current readout from the EG-FET is implemented using a low-noise transimpedance stage based on a precision operational amplifier (LTC2051HV, Analog Devices, Wilmington, MA, USA). The drain–source current is converted into a proportional voltage through a feedback network, followed by bandwidth-limiting (RC filtering) to suppress high-frequency noise and improve measurement stability. The conditioned analog signal is routed to the MCU’s ADC for digitization. Component selection and filtering are designed to balance current sensitivity, dynamic range, and response time.

#### 2.3.2. Power Supply Module

The system supports regulated on-board power for mixed-signal operation. External input power is converted to the required rails using low-dropout regulators (AMS1117, Advanced Monolithic Systems, Livermore, CA, USA), with local decoupling and filtering applied near the analog front-end and the digital core to reduce supply ripple coupling into the measurement path. A dedicated reference source (REF3030, Texas Instruments, Dallas, TX, USA) provides a stable voltage reference for ADC-related measurements and bias stability where applicable.

#### 2.3.3. MCU Module

The MCU (GD32F103C8T6, GigaDevice, Beijing, China) serves as the central controller. It manages sampling timing, ADC acquisition, basic data processing, and communication. Standard peripheral interfaces (UART, SPI, and I^2^C as required) support connectivity to external modules and system expansion. The firmware also handles system initialization and fault recovery through reset/monitoring circuitry.

#### 2.3.4. USB-to-Serial Communication Module

Data transfer to the host computer is implemented using a USB-to-UART bridge (PL2303, Prolific Technology, Shenzhen, China). This module provides a reliable physical and protocol interface for real-time streaming of sampled data and for instrument configuration during testing and debugging.

#### 2.3.5. Auxiliary Modules

To improve measurement robustness and usability, the system includes supporting circuits such as a precision voltage reference, power/reset supervision, and status indicators. Where required, a signal-injection/simulation path is provided for channel verification and calibration during development. These modules ensure repeatable operation and facilitate troubleshooting without altering the sensing interface.

### 2.4. Software Design

#### 2.4.1. Embedded-Software Architecture

The embedded software implements a deterministic acquisition–processing–transmission pipeline, governed by a state machine. Initialization configures the system clock, critical peripherals (GPIO, interrupts), and the analog-to-digital converter (ADC) with predefined sampling parameters. In the main loop, multi-channel voltage sampling is triggered at fixed intervals. The raw ADC samples are processed through a cascade of a median filter and a moving-average filter to suppress noise, then scaled to physical units using a pre-stored calibration factor. The formatted results are packed into application-layer frames and streamed to the host via a DMA-based UART interface, completing the local sensing-to-reporting sequence.

#### 2.4.2. Host-Software Design

A host application, developed on the platform (Visual Studio 2022, Version 17.9.6), provides communication control, data visualization, and storage. A dedicated communication module manages the serial-port link, receives and parses the embedded data frames, and maintains bidirectional command-data exchange. The graphical interface consolidates real-time waveform display, historical trace review, and system-status monitoring. Acquired data are validated, timestamped, and persisted either to flat files or a local database, enabling offline analysis and report generation. Together, these modules form an integrated PC-side platform for instrument operation and data management.

### 2.5. Biofunctionalization of the EG-FET Sensor

#### 2.5.1. Electrode Preparation

This study employed a 3-mercaptopropionic acid (MPA) self-assembled monolayer method to biofunctionalize the screen-printed gold working electrode of the EG-FET sensor for immobilizing *E. coli* O157:H7 monoclonal antibodies. A schematic diagram of the process is shown below. All chemical modification steps—including MPA self-assembly, EDC/NHS activation, antibody immobilization, and BSA blocking—were performed simultaneously on the entire three-electrode system (working, counter, and reference electrodes). The specific steps were as follows: First, the screen-printed gold electrode was sequentially cleaned via ultrasonication in anhydrous ethanol and ultrapure water, followed by drying under a stream of nitrogen to ensure a clean electrode surface. The pre-treated gold electrode was then immersed in a 1.25% MPA solution in ethanol and reacted in the dark at room temperature for 18 h. Subsequently, the electrode was removed and thoroughly rinsed with anhydrous ethanol to remove physically adsorbed MPA molecules, resulting in a dense carboxyl-terminated monolayer on the gold surface. The MPA-modified gold electrode was then placed in an aqueous solution containing 100 mM EDC and 20 mM NHS for 1 h to activate the terminal carboxyl groups of MPA, forming reactive esters. Following this, the electrode was gently rinsed with PBS buffer (pH 7.4), each corresponding solution (e.g., 100 µg/mL *E. coli* O157:H7 monoclonal antibody solution) was precisely dispensed as a 30 µL droplet onto the electrode region using a micropipette, ensuring the droplet completely covers the working electrode. The electrode was then incubated in a humid chamber at 37 °C for 2 h. Under these conditions, the amino groups of the antibody molecules covalently couple with the activated carboxyl groups, achieving stable immobilization of the antibodies on the electrode surface. After the reaction, unbound antibodies were removed by rinsing with PBS buffer. To minimize nonspecific adsorption, the electrode was incubated in a 1% (*w*/*v*) bovine serum albumin (BSA) solution for 1 h for blocking. Finally, the prepared electrodes were stored at 4 °C for later use.

#### 2.5.2. Electrode Characterization

To verify the successful progress of the antibody immobilization process, the electrodes were characterized at each modification stage using cyclic voltammetry (CV) and electrochemical impedance spectroscopy (EIS). All electrochemical measurements were performed on a PalmSens3 electrochemical workstation using a commercially available, integrated screen-printed three-electrode system. This system consists of a gold working electrode, a gold counter electrode, and a screen-printed Ag/AgCl reference electrode. The integrated design ensures that all experiments are conducted on an entirely consistent hardware platform, guaranteeing high comparability of data throughout the entire modification process. Although the gold counter electrode is not completely inert under extreme potentials, its performance remains stable within the limited potential window employed in this study (−0.2 V to +0.6 V vs. Ag/Agcl) and in near-neutral PBS electrolyte, allowing for accurate measurements using the reversible [Fe(CN)_6_]^3−^/^4−^ redox probe.

**Figure 1:**
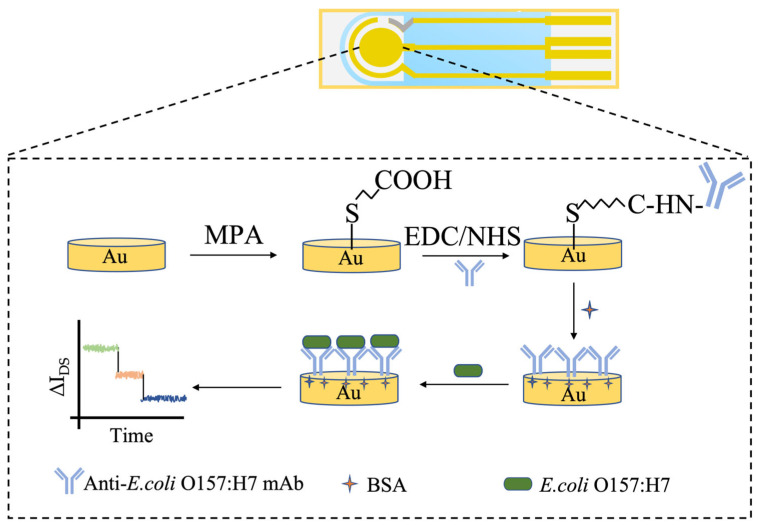
Schematic of the Antibody Immobilization Principle Based on the MPA Method.

The specific procedure is illustrated in Figure 1. Characterization System: Characterization was performed in a solution containing [Fe(CN)_6_]^3−^/^4−^ (0.005 mol/L). Electrodes at different modification stages were tested in the following sequence: bare gold electrode (GE); gold electrode modified with the MPA self-assembled monolayer (GE-MPA); electrode after monoclonal antibody immobilization (GE-MPA-mAb); and the final immunosensor after BSA blocking (GE-MPA-mAb-BSA).

CV measurements were performed using a screen-printed three-electrode system: a gold working electrode, a gold counter electrode, and a screen-printed Ag/AgCl reference electrode. CV scans were initiated after equilibration at open-circuit potential, with a potential range from −0.2 V to +0.6 V (vs. the integrated Ag/AgCl), a scan rate of 50 mV/s, and a potential step of 0.01 V. After each modification step, the stable voltammogram from the third cycle out of ten consecutive scans was recorded.

EIS measurements were conducted in the same electrolyte and electrode system. Prior to the frequency sweep, the system was equilibrated until the open-circuit potential drift fell below 0.5 mV/s The measurement was performed at a DC bias potential of 0 V with a superimposed sinusoidal AC perturbation of 5 mV amplitude. The impedance spectrum was acquired over a frequency range of 0.1 Hz to 100 kHz, with data points collected logarithmically across this range. The Nyquist plot for each modification-stage electrode was recorded at its respective stable open-circuit potential.

### 2.6. Preliminary Detection and Data Analysis

All electrical measurements were conducted at room temperature inside an electromagnetic shielding chamber to ensure signal stability. The functionalized sensor was connected to the test system, with its extended gate—the screen-printed gold electrode—serving as the sensing terminal. The sample solution was applied dropwise to its surface, followed by setting the electrical parameters and data acquisition.

#### 2.6.1. Bias Setting and Signal Monitoring Method

The gate voltage (V_GS_) was fixed near a predetermined threshold voltage (V_th_ ≈ 1.3 V), while the source-drain voltage (V_DS_) was fixed at approximately 1 V to ensure the transistor operated in the linear region. This operating point was selected to balance sensitivity and practicality: it provides high transconductance near Vth for effective signal transduction, while the linear region operation offers greater current stability and lower susceptibility to noise compared to the subthreshold regime, ensuring reliable and reproducible measurements. The source-drain current (I_DS_) was monitored and recorded in real-time as a function of time. When the target analyte binds to the antibodies on the electrode surface, the resulting change in interfacial potential is coupled through the extended gate to the FET channel, thereby modulating I_DS_. The relative change in I_DS_ (ΔI_DS_ = I_DS_ − I_0_, where I_0_ is the baseline current) was used as the signal output for quantitative analysis.

#### 2.6.2. Sensitivity Testing

The *E. coli* O157:H7 bacterial stock was serially diluted with PBS buffer to prepare samples at five concentration points: 10^2^, 10^4^, 10^6^, 10^8^, and 10^10^ CFU/mL. Following an order from low to high concentration, 50 µL of each bacterial dilution was successively applied to the sensing area. After each application, I_DS_ was continuously monitored until it reached a stable plateau (typically within 5–10 min). All measurements were performed in triplicate. For each concentration measurement, a newly prepared electrode was used. Real-time drain–source current responses curves were plotted using the current data from a 30-s window after signal stabilization for each sample. A standard calibration curve was plotted with the logarithm of bacterial concentration on the *x*-axis and the corresponding ΔI_DS_ on the *y*-axis. The limit of detection (LOD) for the sensor was calculated based on this curve.

#### 2.6.3. Specificity Evaluation of the Sensor

To validate the specific recognition capability of the fabricated sensor toward *E. coli* O157:H7, this experiment selected three common foodborne interfering bacterial strains for control testing, including: *Staphylococcus aureus* (SA), *Salmonella typhimurium* (ST), and *Listeria monocytogenes* (LM). The experimental setup was as follows: Sensor responses were recorded for *E. coli* O157:H7, SA, ST, and LM bacterial solutions, as well as for a PBS blank control. Each sample was tested in triplicate. The concentration of *E. coli* O157:H7 was 10^5^ CFU/mL, while the concentrations of the interfering agents were maintained at least 10-fold higher than that of the target bacterium. All other reaction and detection conditions remained identical. The relative change in drain-source current (ΔI_DS_) was recorded, and the average values along with standard deviations for each group were calculated. The response signals of the target bacterium were then compared against those of the interfering strains.

## 3. Results

### 3.1. Integrated EG-FET Sensing System: Architecture and System-Level Specifications

**Figure 2:**
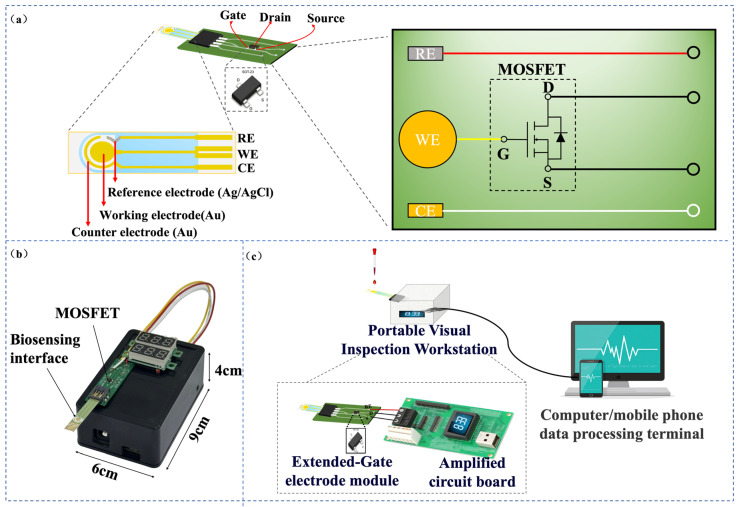
(**a**) Schematic of the extended-gate electrode module. The red signal line is connected to the reference electrode, the yellow signal line extends the gate of the MOSFET to the working electrode (a screen-printed Au electrode), and the black signal line connects the source and drain terminals of the MOSFET to the amplification circuit for current signal amplification. The counter electrode functions as a backup electrode (**b**) Photograph and dimensions of the fabricated device. (**c**) Schematic diagram illustrating the overall system architecture.

The proposed EG-FET integrated sensing system is implemented as a compact, three-stage chain for on-site biosensing (Figure 2a–c), combines (i) an extended-gate sensing module, (ii) a low-noise analog front end for current readout, and (iii) an MCU-based acquisition and host-side analysis module. The key specifications of the platform are summarized in Table 1, encompassing the system overview, power and signal chain performance, noise characteristics, and communication interface stability.

The sensing function originates from the extended-gate module, which is realized using a screen-printed Au electrode (Figure 2a). This electrode provides a defined, chemically addressable surface for immobilizing biorecognition probes and is electrically coupled to the MOSFET gate via an external lead. This design allows biorecognition to occur at the solid–liquid interface while isolating the transistor core from direct electrolyte exposure. Upon target binding, the perturbation in interfacial charge distribution and surface potential is transferred to the MOSFET gate as an effective bias shift. This modulation alters the channel conduction, resulting in a measurable drain–source current response under a fixed operating point, thereby transducing a biological event into an electrical signal.

#### 3.1.1. Hardware Implementation

**Figure 3:**
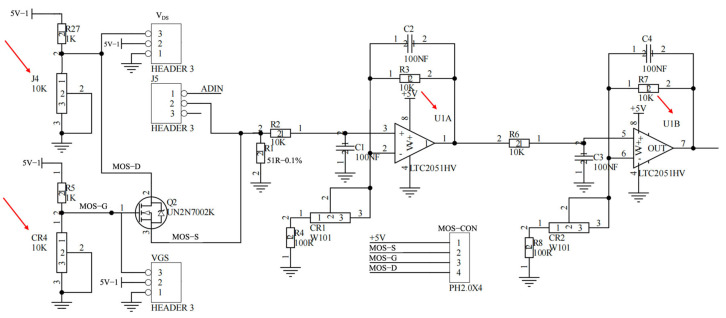
Current Sampling and Signal Conditioning Module.

**Figure 4:**
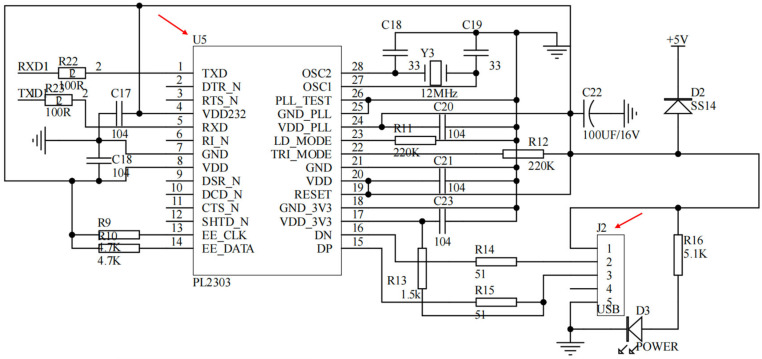
USB-to-TTL module.

**Figure 5:**
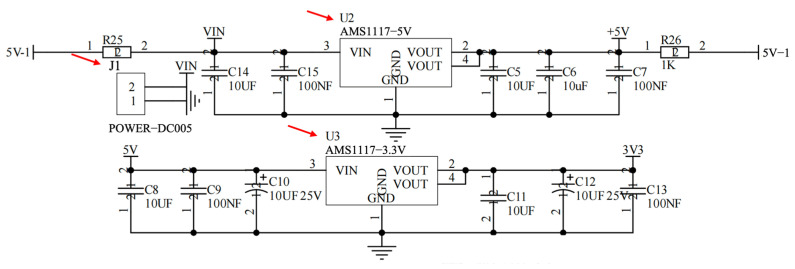
Power supply module.

**Figure 6:**
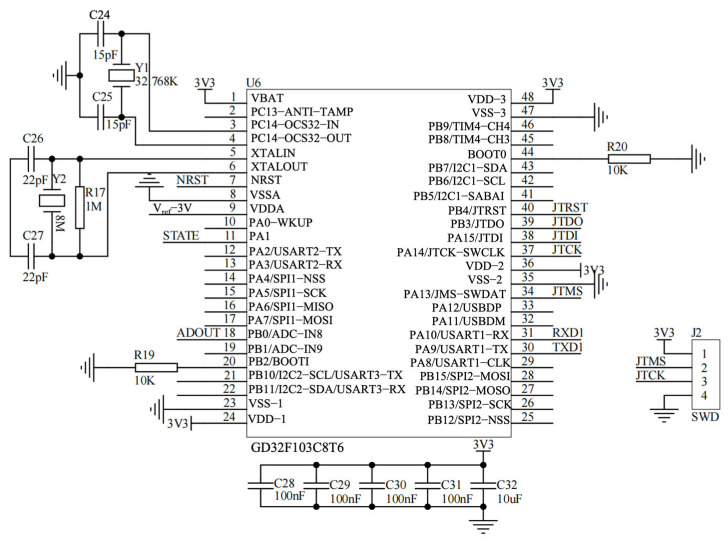
MCU module.

**Figure 7:**
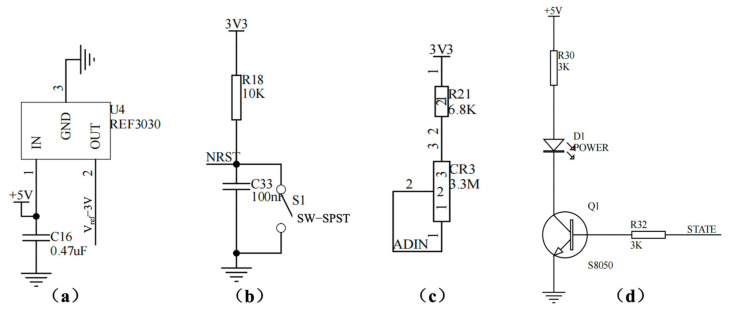
Schematics of auxiliary circuit modules: (**a**) reference voltage circuit, (**b**) reset circuit, (**c**) demonstration/signal simulation circuit, (**d**) status indication circuit.

The entire system can be viewed as a modular data-acquisition chain with a clear signal path. The process begins with the Sensor Module (the screen-printed electrode), where current variations (ΔI_DS_) induced by biorecognition events are extracted. This weak current is first directed to the Analog Front-End Module, which serves two core functions: providing precisely adjustable static bias voltages (V_DS_ and V_GS_) for the sensing FET, and converting the current signal into a voltage signal suitable for sampling via a low-noise, programmable-gain two-stage amplification circuit with integrated filtering. The conditioned analog voltage is then fed into the Acquisition and Control Module centered around the MCU, where it is digitized by the internal ADC. The MCU manages data packaging, basic processing, and auxiliary circuits such as a precision voltage reference and reset control. The digitized data is transmitted to the host computer via the stable Communication Interface Module (a USB-to-UART bridge). All analog and digital circuits are supported by a dedicated Power Module that features multiple inputs and cascaded regulation to deliver a clean and stable power supply, which is fundamental for high-precision weak-signal measurement. Further detailed hardware design schematics are provided in Figure 3, Figure 4, Figure 5, Figure 6 and Figure 7.

##### Analog Front End and Bias Control

The MOSFET operating point is established by independently setting V_DS_ and V_GS_ using on-board adjustment nodes (J4 and CR4), enabling stable biasing prior to signal acquisition. The sensor current is conditioned by a low-noise, two-stage amplification circuit based on a precision dual operational amplifier (LTC2051HV), as shown in Figure 3. The first stage converts the weak sensor output into a voltage signal with adjustable gain, while the second stage provides additional amplification and conditioning. Gain trimming is performed through dedicated potentiometers (CR1 and CR2), allowing the readout range to be matched to the expected signal amplitude. RC networks are incorporated for bandwidth shaping to suppress high-frequency noise and improve measurement stability under liquid-phase operation.

##### Data Interface and Power Delivery

The USB-to-Serial communication module adopted in this study utilized the PL2303 as its core chip, establishing a stable communication bridge between the computer’s USB interface and the microcontroller’s UART. As shown in Figure 4, the module performed the complete conversion from USB differential signals to TTL-level serial signals through the PL2303’s built-in USB protocol processor and UART controller. An integrated LDO voltage regulator directly provided the 3.3 V operating voltage, and a 12 MHz crystal oscillator ensured precise communication timing. The USB interface employed a standard Type-A connector, with the D+/D− signal lines optimized for signal integrity via series matching resistors and ESD protection devices. The TTL side directly provided TXD and RXD signal lines, supporting 3.3 V/5 V level-compatible communication. The entire circuit effectively suppressed power supply noise through a multi-stage decoupling capacitor network, and the differential signal lines strictly followed equal-length routing rules, reflecting careful design considerations for signal integrity and anti-interference capability, thereby providing a stable and reliable host computer communication channel for the system.

Communication with the host computer is implemented through a USB-to-UART bridge (PL2303), which converts the USB interface to TTL-level serial signals for real-time streaming and device debugging (Figure 4). To ensure measurement robustness under different deployment conditions, the power subsystem supports both USB and external-adapter inputs (Figure 5). The USB path provides a 5 V supply with reverse-polarity protection, whereas the external input accepts a wide voltage range and is regulated in a cascaded manner to generate stable intermediate and logic rails (e.g., 5 V followed by 3.3 V). Local decoupling and staged regulation are used to reduce supply ripple coupling into the analog front end, which is critical for weak-current measurements.

##### MCU and Auxiliary Circuits

The GD32F103C8T6 MCU coordinates sampling, data framing, and communication (Figure 6). The conditioned analog signal is routed to a dedicated ADC input, and a stable reference is provided to improve conversion repeatability. Auxiliary circuits are included to support long-term operation and testability (Figure 7), including a precision reference source (REF3030) for low-drift voltage referencing, an automatic/manual reset network for fault recovery, a programmable current-injection path for functional verification and calibration during development, and status indicators for operational monitoring. Together, these modules provide a complete hardware environment for stable readout, data acquisition, and host communication within a compact prototype.

#### 3.1.2. Software Implementation

**Figure 8:**
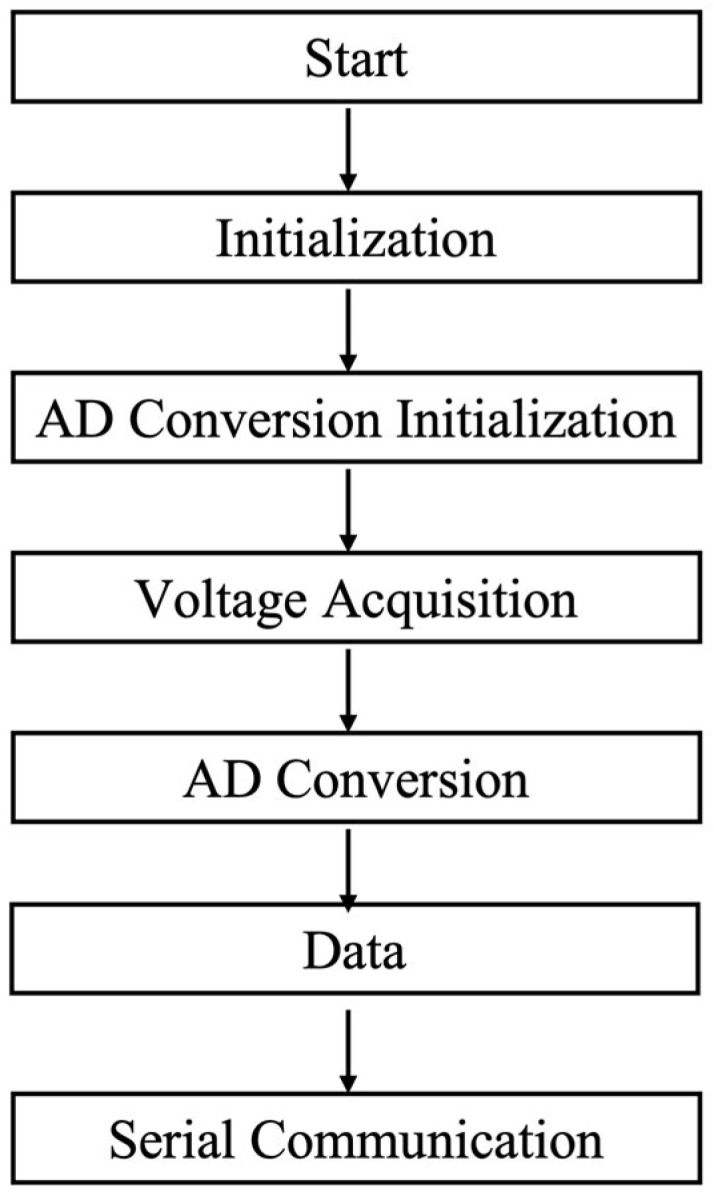
Embedded-Software architecture structure design.

The software stack follows a two-layer architecture comprising embedded firmware and host-side software. The embedded program executes a structured acquisition pipeline (Figure 8): after power-on initialization (clock, GPIO, ADC, and UART), the MCU performs periodic sampling, applies lightweight denoising (median filtering followed by moving-average smoothing), and converts raw ADC codes into engineering values using calibration coefficients. Processed data are then packaged into predefined frames and transmitted over UART to ensure consistent parsing on the host side.

Host-side software was developed in Visual Studio and provides serial-port management, real-time display of current signals, and data logging. The interface separates configuration functions (port selection and connection control) from live readout, and it supports continuous monitoring and storage of measurement records for subsequent quantitative analysis. This coordinated firmware–software workflow enables end-to-end operation from electrical signal acquisition at the EG-FET front end to visualization and archiving at the host interface, forming a complete and reproducible measurement loop for the EG-FET integrated sensing system.

### 3.2. Performance Characterization of the Biofunctionalized EG-FET

**Figure 9:**
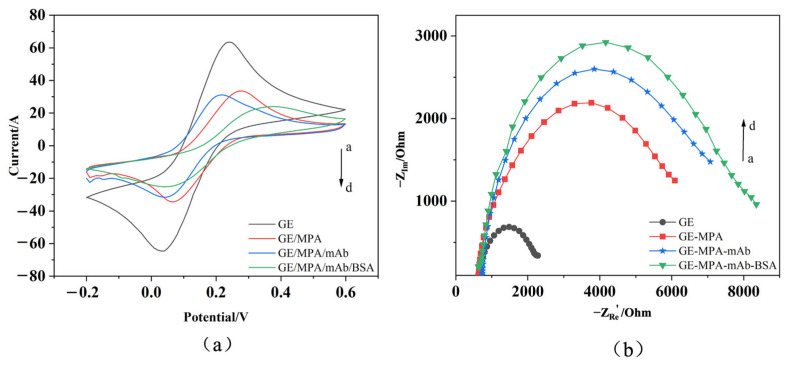
Electrode characterization by (**a**) cyclic voltammetry and (**b**) electrochemical impedance spectroscopy during stepwise biofunctionalization.

The stepwise fabrication of the extended-gate immunosensing interface was verified by cyclic voltammetry (CV) and electrochemical impedance spectroscopy (EIS) using the [Fe(CN)_6_]^3−^/^4−^ redox probe (Figure 9). CV provides a direct readout of interfacial electron-transfer accessibility, whereas EIS quantifies the corresponding charge-transfer resistance and is sensitive to surface coverage and insulating layers formed during modification.

As shown in Figure 9a, the bare Au electrode exhibits well-defined and quasi-reversible redox peaks with the highest peak currents (curve a). After formation of the MPA self-assembled monolayer, the peak currents decrease markedly (curve b), consistent with the introduction of a compact thiol layer (Au–S bonding) that partially blocks electron transfer. Subsequent EDC/NHS activation followed by immobilization of anti-*E. coli* O157:H7 monoclonal antibodies further suppresses the peak currents (curve c), indicating increased interfacial steric and electrostatic hindrance arising from the protein layer. After BSA blocking, the redox response is minimized (curve d), reflecting additional passivation of remaining active sites and reduced nonspecific adsorption.

The EIS results corroborate the CV trends. In the Nyquist plots (Figure 9b), the bare electrode shows the smallest semicircle, corresponding to the lowest charge-transfer resistance Rct (curve a). MPA modification increases the semicircle diameter (curve b), evidencing hindered interfacial electron transfer. The antibody layer and subsequent BSA blocking lead to a further monotonic increase in the semicircle diameter (curves c and d), consistent with progressive growth of an interfacial barrier. The agreement between CV and EIS confirms successful layer-by-layer construction of the biofunctionalized extended gate, providing a validated sensing interface for subsequent bacterial detection experiments.

### 3.3. Detection Performance for E. coli O157:H7

#### 3.3.1. Real-Time Current Response

**Figure 10:**
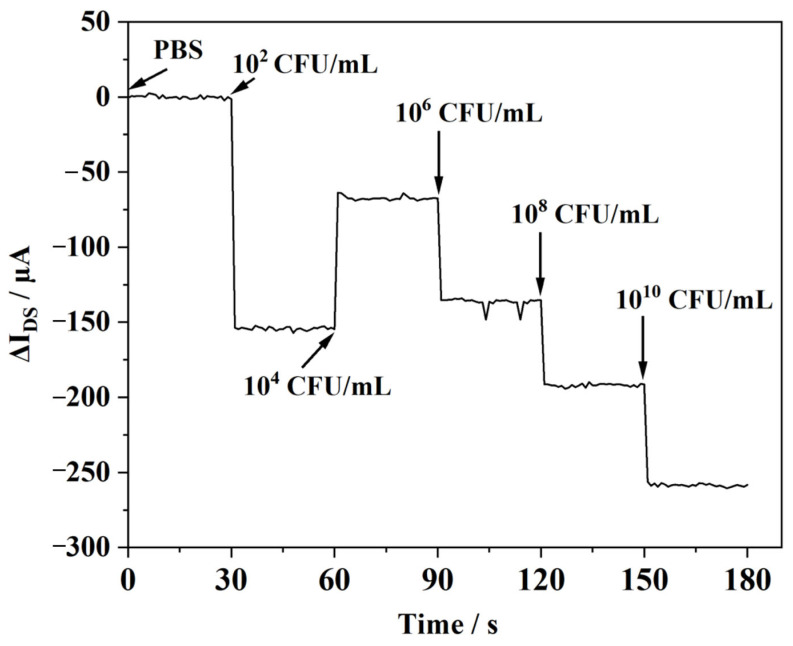
Real-time drain–source current responses curves of the EG-FET to *E. coli* O157:H7 suspensions at different bacterial concentrations.

The dynamic response characteristics of the EG-FET integrated sensing system were evaluated using *E. coli* O157:H7 suspensions at five concentration points: 10^2^, 10^4^, 10^6^, 10^8^, and 10^10^ CFU/mL. Representative real-time current traces are shown in Figure 10. Under fixed bias conditions, the drain–source current exhibited a concentration-dependent decrease as the bacterial concentration increased. A monotonic trend was observed over 10^4^ to 10^10^ CFU/mL, indicating that the sensor output is governed by bacteria-induced perturbations of the interfacial potential at the biofunctionalized extended gate, which are transduced into modulation of channel conductance. In this regime, higher concentrations are expected to produce a larger surface coverage and a correspondingly stronger effective gate-voltage shift, yielding a more pronounced current change.

Notably, the response at the lowest tested concentration (10^2^ CFU/mL) deviated from the overall trend, showing an anomalous signal. This is likely because this concentration is near the current system’s detection limit, where the signal may be interfered with by baseline noise, nonspecific adsorption, or unstable binding kinetics at low concentrations. To resolve this low-concentration behavior, subsequent experiments will emphasize replicate measurements and variance analysis, together with optimization of the recognition-layer assembly and baseline handling (including drift compensation) to improve precision near the detection boundary.

#### 3.3.2. Sensitivity and Calibration Curve

**Figure 11:**
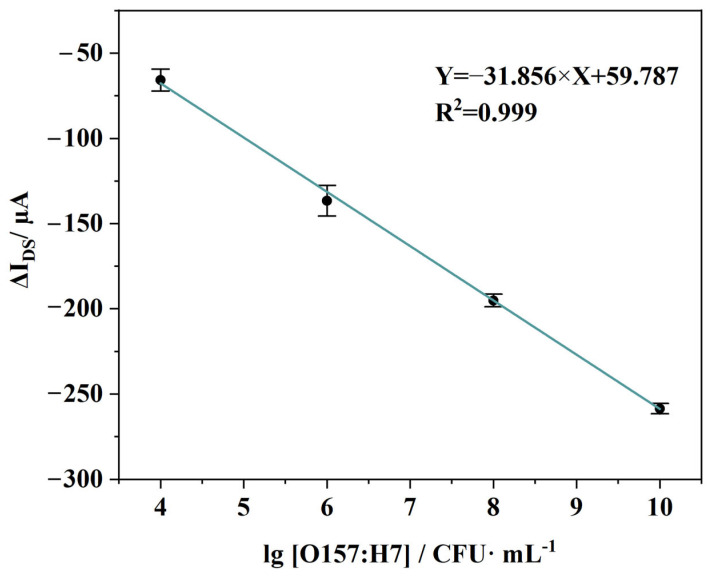
Calibration curve of the EG-FET sensor for *E. coli* O157:H7 detection.

A calibration curve was constructed by extracting the steady-state drain–source current from the real-time traces and converting it to the current response ΔI_DS_, relative to the baseline. ΔI_DS_ was then plotted against the logarithm of *E. coli* O157:H7 concentration (Figure 11). All measurements were performed in triplicate. The results indicate a good linear relationship between ΔI_DS_ and log C within the concentration range of 10^4^ to 10^10^ CFU/mL. The linear regression equation is ΔI_DS_ = −31.856 log C + 59.787, with a correlation coefficient (R^2^) of 0.999. The relative coefficients of variation (in absolute terms) for the four measurement points range from 1.2% to 9.8% To avoid subjective data handling, the lowest-concentration point is not removed by manual exclusion; instead, it is treated separately as a near-threshold regime that is used to assess detection onset rather than to define the primary quantitative range.

The practical limit of detection (LOD) is conservatively defined as the lowest concentration reliably quantified within the validated linear range, which is 10^4^ CFU/mL. This definition is based on the experimental observation that responses at concentrations below this threshold were unstable and irreproducible. A statistical estimate using the 3σ/|S| criterion (where σ is the standard deviation of blank measurements, and |S| is the absolute slope) yields a theoretical LOD of approximately 9.55 CFU/mL. This value reflects the high intrinsic sensitivity of the sensing principle but represents an extrapolation beyond the experimentally verified stable regime. It should therefore be interpreted as an indicator of potential sensitivity, not the current operational detection capability.

To further bridge the gap between the theoretical sensitivity and the experimentally verified quantitative range, future work will include additional repeated measurements at concentrations approaching and below the theoretical LOD of 9.55 CFU/mL. These studies aim to systematically assess the reproducibility, accuracy, and precision of the sensor response in this low-concentration regime, thereby determining whether the statistically derived LOD can be experimentally validated as a reliable detection threshold.

#### 3.3.3. Results of Specificity Evaluation

**Figure 12:**
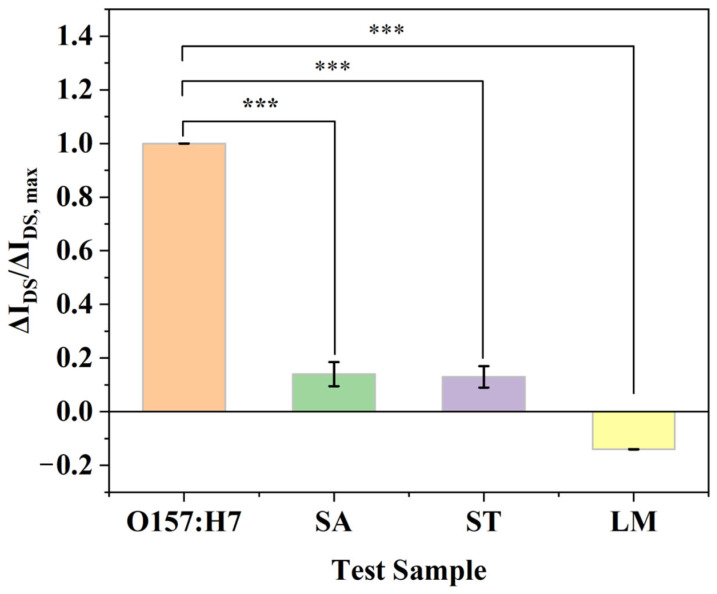
Specific detection of *E. coli* O157:H7 using antibody-functionalized EG-FET.

Specificity evaluation is a crucial step in biosensing detection. After confirming that the EG-FET could sensitively detect *E. coli* O157:H7, we further selected three closely related common foodborne pathogens—*Staphylococcus aureus* (SA), *Salmonella typhimurium* (ST), and *Listeria monocytogenes* (LM)—as interferents to assess the selective recognition capability of the biosensor. In the experiment, the target *E. coli* O157:H7 concentration was set at 10^5^ CFU/mL, while the concentration of each interferent was 10^6^ CFU/mL, representing a more than 10-fold excess relative to the target. The results are shown in Figure 12. Using the blank response as the baseline, the response induced by the inactivated whole-cell protein of the target was defined as ΔImax, and the responses caused by the three interferents were denoted as ΔI. The maximum ratio of ΔI to ΔImax reached only 13%, indicating that the target response was approximately 7.7 times higher than those of the interferents. Hence, the influence of other bacterial factors on the detection outcome is negligible, demonstrating that this monoclonal-antibody-modified sensor exhibits excellent selectivity toward *E. coli* O157:H7.

It should be emphasized that although LM exhibited an opposite response trend, its absolute signal magnitude (ΔI_DS_/ΔI_DS,max_ ≈ 13%) was significantly lower than the target response (defined as 100%). This fact further confirms the high specificity of the monoclonal antibody modified on the sensor for *E. coli* O157:H7. The weak inverse signal induced by LM is more likely attributable to a unique physicochemical perturbation at the sensing interface caused by its surface properties, rather than to cross-reactivity with the antibody. This interesting observation provides a valuable direction for future research into the complex interactions between bacterial surface characteristics and biosensor interfaces. A statistically significant difference was observed between the target and non-specific groups (*p* < 0.001, denoted by *** in Figure 12).

## 4. Discussion

This study demonstrates an integrated EG-FET biosensing system for the detection of *E. coli* O157:H7, the performance of which has been verified under laboratory conditions. To fully interpret the significance of these results and clarify the position of this work, it is necessary to discuss them within a broader research context and application prospects. First, the observed sensing signal originates from interfacial potential changes induced by bacterial capture, but this process is significantly constrained by the Debye screening effect. When the concentration of PBS increases from 0.1 × 10^−3^ mol/L to 10 × 10^−3^ mol/L, the Debye length decreases from 7.0 nm to 0.7 nm. Typically, the length of receptor molecules immobilized on the device ranges from 5 to 15 nm, and their distance from the sensing surface is between 2 and 12 nm [28,29]. Consequently, the size of target molecules and their distance from the device surface often exceed the Debye length of the FET, leading to partial charge shielding of the target molecules and thereby reducing detection sensitivity. This provides a theoretical basis for understanding the current sensitivity limits and indicates a future pathway for systematically optimizing buffer ionic strength to improve the signal-to-noise ratio and detection limit.

In terms of innovation, the core contribution of this work lies in the realization of an EG-FET integrated solution that balances performance, cost, and practicality. Unlike many conventional designs, the extended-gate architecture adopted in this study physically separates the sensitive interface from the transistor core. This not only enhances device robustness and flexibility in interface modification but is also key to achieving low-cost, disposable extended gates. Particularly important is our preliminary cost analysis, which shows that the hardware cost of the entire setup can be controlled at approximately USD 5.6, while the cost of the screen-printed gold electrode consumed per test is about USD 1.4. This establishes a highly competitive foundation for subsequent productization. In the future, exploring lower-cost substrate materials such as carbon electrodes will be a major focus for further reducing the cost per test and promoting practical application.

Nevertheless, we must objectively acknowledge that a gap remains between the current stage of this research and the goal of “field applicability.” To date, all experiments have been conducted under controlled laboratory conditions, lacking performance validation in real-world environments such as battery-powered operation, temperature and humidity fluctuations, or long-term continuous operation. Therefore, statements regarding the stability and robustness of the system for field deployment remain somewhat speculative at this stage. This clearly delineates the directions that future research must encompass: namely, long-term stability testing, environmental drift characterization, and validation with actual complex samples. Only through such rigorous evaluation can the current high-performance laboratory prototype be transformed into a reliable field device. Furthermore, to bridge the gap between the sensor’s reliable quantitative detection range (10^4^–10^10^ CFU/mL) and the stringent regulatory thresholds typically required in practical applications (e.g., 10^2^–10^3^ CFU/mL in the field of food safety), future work may need to introduce a sample pre-concentration step prior to detection.

In summary, this study not only verifies the feasibility of the integrated EG-FET system for pathogen detection, but also, through in-depth analysis of its operating mechanism, cost structure, and application limitations, outlines a clear roadmap for technological development. Future efforts should focus on overcoming performance bottlenecks via interfacial physicochemical optimization, reducing manufacturing costs through material and process innovation, and ensuring reliability in practical scenarios via stringent environmental testing, thereby ultimately achieving the transition from a laboratory concept to a field-ready solution.

## 5. Conclusions

In this work, we developed a compact EG-FET integrated sensing system that combines an extended-gate sensing electrode, a low-noise readout/acquisition module, and host-side visualization software into an end-to-end prototype for on-site biosensing. By integrating bias control, weak-current conditioning, digital sampling, and real-time data display within a single system, the proposed implementation reduces dependence on benchtop instrumentation and provides a practical engineering route toward deployable FET-based detection. By covalently immobilizing *E. coli* O157:H7 monoclonal antibodies on the Au extended gate, we constructed a target-specific immunorecognition interface. Measurements in buffer demonstrated a concentration-dependent electrical response, with a linear calibration behavior validated over 10^4^–10^10^ CFU/mL, and a theoretical limit of detection (LOD) of 9.55 CFU/mL, supporting quantitative detection within this range. The specificity experiments further confirmed that the signal response to the target bacterium (*E. coli* O157:H7) (ΔI_DS_/ΔI_DS_, _max_ = 100%) was significantly higher than that to non-target strains, with a highly statistically significant difference between the two groups (*p* < 0.001), demonstrating the high specificity of the constructed immunorecognition interface.

This study primarily establishes system feasibility and integration methodology. Future work will focus on the following key directions: optimizing buffer ionic strength to mitigate the Debye screening effect; enhancing the system’s low-concentration precision, long-term stability, and drift resistance; and conducting rigorous robustness evaluation and application-oriented testing. These efforts are directed toward advancing the system toward practical deployment in on-site diagnostic scenarios such as food safety monitoring.

## Figures and Tables

**Table 1:** System-level specifications of the integrated EG-FET biosensing platform.

Category	Parameter	Specification/Value	Condition/Note
System overview	Device Footprint	7.5 cm × 4.5 cm	Main PCB dimensions
	Supply Voltage	5 V(USB)/5–18 V (DC adapter)	Dual-input design
Power and signal chain	Operating Current	750 μA (Typical)/1 mA (Maximum) per amplifier.	Includes all active components
	Sampling Rate	Sampling rate: 800–1000 kHz (raw)/Output interval: 300 ms/point (~3.33 Hz effective)	Set by MCU firmware
Noise and resolution	Output Baseline Drift	1.5μV_P-P_ (0.01 Hz to 10 Hz Typical)	Short-term stability at fixed bias
	ADC Resolution	12-bit	GD32F103C8T6 embedded ADC
	Gain-Bandwidth Product (GBP)	3 MHz	Typical value of analog front-end op-amp (LTC2051HV)
Communication	Interface	USB to UART Bridge	Via PL2303 bridge
	Baud Rate	Up to 921,600 bps	Configurable; 115,200 bps typical
	Data Streaming Stability	Error-free for >1 h	Continuous operation test

## Data Availability

The data that support the findings of this study are available from the corresponding authors upon reasonable request.
